# MXene (Ti_3_C_2_T_x_)-Embedded Nanocomposite Hydrogels for Biomedical Applications: A Review

**DOI:** 10.3390/ma15051666

**Published:** 2022-02-23

**Authors:** Fouad Damiri, Md. Habibur Rahman, Mehrukh Zehravi, Aeshah A. Awaji, Mohammed Z. Nasrullah, Heba A. Gad, Mutasem Z. Bani-Fwaz, Rajender S. Varma, Mousa O. Germoush, Hamdan S. Al-malky, Amany A. Sayed, Satish Rojekar, Mohamed M. Abdel-Daim, Mohammed Berrada

**Affiliations:** 1 Labortory of Biomolecules and Organic Synthesis (BioSynthO), Department of Chemistry, Faculty of Sciences Ben M’Sick, University Hassan II of Casablanca, Casablanca 20000, Morocco; berrada_moh@hotmail.com; 2 Department of Global Medical Science, Wonju College of Medicine, Yonsei University, Wonju 26426, Gangwon, Korea; 3 Department of Clinical Pharmacy Girls Section, Prince Sattam Bin Abdul Aziz University Alkharj, Alkharj 11942, Saudi Arabia; mahrukh.zehravi@hotmail.com; 4 Department of Biology, Faculty of Science, University College of Taymaa, University of Tabuk, Tabuk 71491, Saudi Arabia; aawaji@ut.edu.sa; 5 Department of Pharmacology and Toxicology, Faculty of Pharmacy, King Abdulaziz University, Jeddah 21589, Saudi Arabia; mnasrullah@bmc.edu.sa; 6 Department of Pharmaceutical Sciences, Pharmacy Program, Batterjee Medical College, Jeddah 21442, Saudi Arabia; h.gad@pharma.asu.edu.eg; 7 Department of Pharmaceutics and Industrial Pharmacy, Faculty of Pharmacy, Ain Shams University, Cairo 11566, Egypt; 8 Department of Chemistry, College of Science, King Khalid University, Abha 61413, Saudi Arabia; mbanifawaz@kku.edu.sa; 9 Regional Centre of Advanced Technologies and Materials, Czech Advanced Technology and Research Institute, Palacky University, Šlechtitelů 27, 783 71 Olomouc, Czech Republic; varma.rajender@epa.gov; 10 Biology Department, College of Science, Jouf University, Sakaka 72388, Saudi Arabia; mogermoush@ju.edu.sa; 11 Regional Drug Information Center, Ministry of Health, Jeddah 21589, Saudi Arabia; hamdan27@hotmail.com; 12 Zoology Department, Faculty of Science, Cairo University, Giza 12613, Egypt; amanyasayed@sci.cu.edu.eg; 13 Department of Pharmaceutical Sciences and Technology, Institute of Chemical Technology, Mumbai 400019, India; rojekarsatish@gmail.com; 14 Pharmacology Department, Faculty of Veterinary Medicine, Suez Canal University, Ismailia 41522, Egypt

**Keywords:** MXenes (Ti_3_C_2_T_x_), nanocomposites, biomedical, nanotechnology, nanomaterials

## Abstract

Polymeric nanocomposites have been outstanding functional materials and have garnered immense attention as sustainable materials to address multi-disciplinary problems. MXenes have emerged as a newer class of 2D materials that produce metallic conductivity upon interaction with hydrophilic species, and their delamination affords monolayer nanoplatelets of a thickness of about one nm and a side size in the micrometer range. Delaminated MXene has a high aspect ratio, making it an alluring nanofiller for multifunctional polymer nanocomposites. Herein, we have classified and discussed the structure, properties and application of major polysaccharide-based electroactive hydrogels (hyaluronic acid (HA), alginate sodium (SA), chitosan (CS) and cellulose) in biomedical applications, starting with the brief historical account of MXene’s development followed by successive discussions on the synthesis methods, structures and properties of nanocomposites encompassing polysaccharides and MXenes, including their biomedical applications, cytotoxicity and biocompatibility aspects. Finally, the MXenes and their utility in the biomedical arena is deliberated with an eye on potential opportunities and challenges anticipated for them in the future, thus promoting their multifaceted applications.

## 1. Introduction

Since the initial determination of graphene in 2004 [1], the layered (2D) structure materials have sparked a lot of curiosity, exhibiting unique chemical and physical properties that are different from those of the their bulk counterparts, with the main objectives being to exploit new 2D materials with interesting properties for unique practical applications [2]. Gogotsi’s group at Drexel university recently developed a vast family of transition metal nitrides and/or carbides, termed MXenes [3], which are also obtainable from other layered compounds, such as Zr_3_Al_3_C_5_ and Mo_2_Ga_2_C. The common formula for MXenes can be transcribed as: M_n+1_X_n_T_x_ (*n* = 1–3) [4], where M is an early transition metal (e.g., Zr, Ti, V, Ta, Nb, or Mo) and X is nitrogen and/or carbon [5]. Such MXenes are usually prepared from their corresponding mass in MAX phase by careful etching of layers of Group IIIA or IVA elements by fluoride-based entities, and therefore their basal faces are frequently finished with surface moieties (T_x_), namely, a mixture of O, F and OH [6,7,8].

Due to their intrinsic metallic conductivity and hydrophilic nature, MXenes exhibit extraordinary physicochemical properties (electronic, magnetic, optical, mechanical and so on) (see Table 1). In terms of ceramics, MAX phases offer enhanced hardness, decreased density and superior corrosion resistance, whereas in terms of metallic materials, they have strong electrical conductivities and good machinability. Due to their distinct characteristics, MAX phases have outstanding difficulties for high-temperature structural applications [9].

There are already around 70 MAX phases known, as well as several solid solutions of these phases, such as the P63/mmc layered hexagonal crystal structure with M layers densely packed and X atoms occupying the octahedral positions, as well as neighboring M_n+1_X_n_ layers interleaved with pure A-layers [5]. Since the M–X link in MAX phases is frequently blended metallic/covalent, while the M–A bond is metallic, mechanically breaking the links between MAX layers is challenging [12].

**Figure 1:**
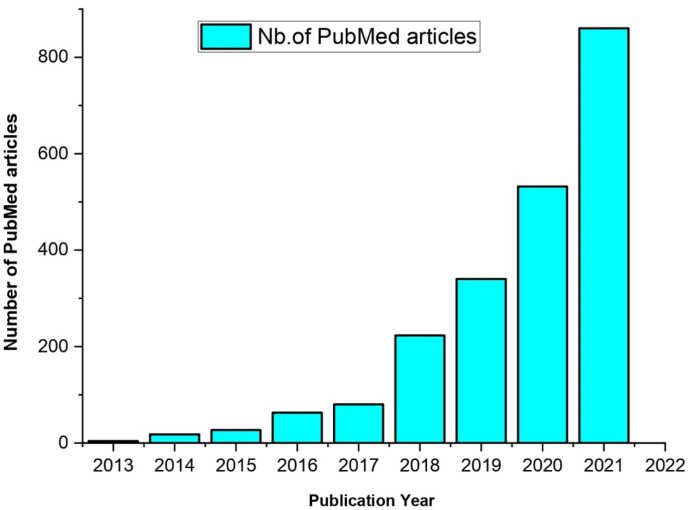
Yearly distribution of scientific articles published on MXenes in PubMed-indexed scientific journals from 2012 to December 2021.

MXenes can be used as inherent active materials and/or carriers of other functional materials for a variety of applications, comprising energy storage and conversion, due to their excellent chemical and structural tunability [13,14], electromagnetic interference shielding [15], sensors [16], biomedical imaging and therapy [10,17], water purification [18], gas separation [19] and catalysis [20], and due to their specific metallic conductivity and the profusion of functionalities on the surface. The incessant growth of the emerging MXenes research is illustrated by a progressive yearly increase of published papers (Figure 1).

Moreover, MXenes are of special relevance in hydrogel-based applications because of their outstanding mechanical strength, extraordinary hydrophilicity and a distinct surface chemistry, which adds a new level of adaptability [21,22]. Furthermore, when MXenes have been introduced into hydrogel systems, they create better characteristics as well as impart interesting potential functions for enhanced performance in a variety of applications [23]. Many novel qualities of the created MXene-based gels are due to the intrinsic properties of the MXenes, the combination of the functions of both MXenes and other elements in the gel matrix, or the interactive effects among them. Consequently, the incorporation of MXenes into hydrogels not only allows the fabrication of MXene-based soft materials with size-dependent properties, but it also significantly improves MXenes’ stability, which is typically a limiting factor in many of their uses. Furthermore, assorted MXene hydrogel derivatives, such as aerogels, may be created using simple procedures, thus increasing the variety of their applications. It is worth noting that there has been an exponential increase in high-value papers pertaining to MXene hydrogels and their variants in the last two years, which makes it a strong competitive field of study. 

Accordingly, the goal of this well-timed review is to stimulate the development of such emergent systems and to broaden the utility range of both hydrogels and MXenes. Until recently, multiple porous MXenes with suitable designs have been built using various synthetic methodologies and implemented for a variety of applications with greatly improved performance. The effect of the synthesis pathway is discussed first to describe MXenes’ structure and stability, followed by a description of their electronic structures, the highly relevant electrical, mechanical, thermal and magnetic properties, and some of the most promising applications, such as energy-linked, biomedical electronic and composite fortifications [24,25]. Finally, based on the existing challenges [26], an imminent inquiry perspective is proposed, with the claim that future theoretical and experimental studies will use this study as a useful reference to deliberate, compare and enhance the MXene-based knowledge [27].

## 2. MAX Phases

**Figure 2:**
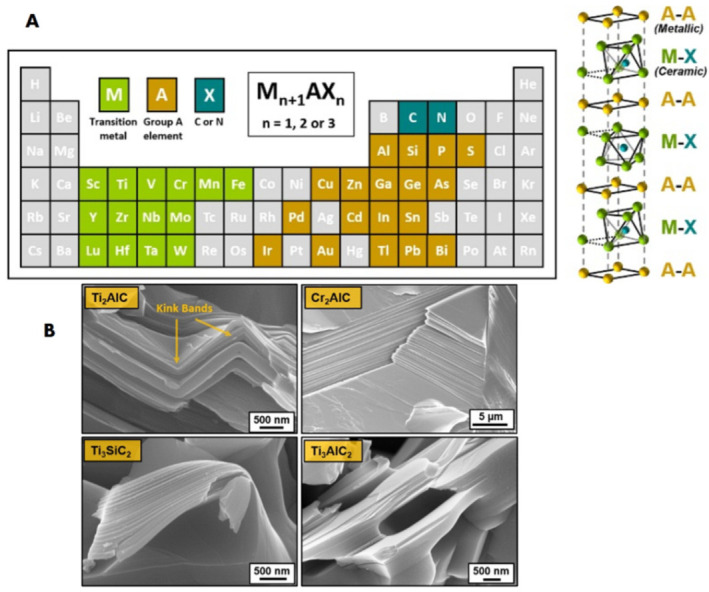
(**A**) MAX phases M_n+1_AX_n_ forming elements. (**B**) SEM pictures of the characteristic layered structure and the mechanical response of Ti_2_AlC, Cr_2_AlC, Ti_3_SiC_2_ and Ti_3_AlC_2_ [39].

The MAX phase is the predecessor to MXene, with a stoichiometry of M_n+1_AX_n_ (Figure 2), with *n* = 1, 2, or 3, where “M” is a d-block transition metal, “A” signifies group 13 and 14 elements (namely Al, Si, Ge, or Sn) and “X” is nitrogen, carbon [25]. The phases have a hexagonal structure (space group P63/mmc) with intercalated layers “M” and “A.” The “X” atoms fill the octahedral spaces left by the “M” elements. There are around 70 recognized MAX phases (such as Ti_2_A_l_C and Ti_3_A_l_C_2_), and new ones (such as the quaternary ordered MAX phases) and comparable materials, namely the “MAB phases”, are continually being found [26].

Their unique features, resulting from an uncommon blending of ceramic and metallic behaviors, have attracted the curiosity of many researchers worldwide. MAX phases are ceramics with high hardness, low density and superb corrosion endurance, whereas metallic materials have strong thermal and electrical conductivities and outstanding processability [28]. Due to their particular properties, MAX phases are capable materials for high-temperature structural appliances (e.g., heating elements, heat exchangers and nozzles), rotating electrical contacts, heating elements and bearings and wear and corrosion protection resources [29]. The following summary in Table 2 discusses the oligolayer MXene composite hydrogel fabrication techniques.

The fundamental bonds are responsible for these innovative properties: with the exception of M-X bonds, which include a combination of metallic, ionic and covalent components, MA bonds are purely metallic. Unlike another 3D layered materials such as transition metal dichalcogenides and graphite, that are connected by weak Van der Waals interactions, MAX phases have sturdy bonds that inhibit severing via shearing or additional mechanical techniques. Thus, the chemical exfoliation method has enabled the production of 2D materials (MXenes) from primary bonded solids (MAX phases) (Figure 2) [38]. 

## 3. Properties of MXene (Ti_3_C_2_T_x_) 

As has been perceived from earlier sections, MXenes can be prepared in a variety of ways, and they can also be incorporated into polymers to change their characteristics. This section discusses the mechanical, electrical and barrier characteristics of MXene/polymer NCs, and their thermal and dimensional stabilities [40].

The strong metallic conductivity of MXenes is one of their most intriguing features. Conductivities of spin-cast Ti_3_C_2_T_z_ films are in the order of 10,000 S/cm^2^ [41]. As a result of embedding MXene in polymers, conductive NCs can be made, which can be employed as safeguards against radio frequency and electromagnetic interference, flexible strain sensors, gas- and molecule-selective sensors and energy conversion and storage applications. Table 3 shows the percolation cut-off, or the volume fraction of MXene necessary to produce a percolating network of conductive channels, for a variety of polymers. Lipatov et al. showed extremely high-breakdown current density in a monolayer of Ti_3_C_2_T_x_, a material in the family of two-dimensional transition metal carbides known as MXenes, which outperforms copper and other base metals [42].

The transition metal in the MAX phase is mainly responsible for the intrinsic characteristics of MXenes. As a result, the bulk of the known magnetic MXenes are based on transition metal magnetic elements such as Mn, MO, Cr, V, Fe, Co and Ni [43,44]. However, MXenes based on nonmagnetic transition metals are expected to be magnetic, and their electronic, optical and magnetic characteristics can be influenced by surface termination groups, defects and synthesis methods [45]. In particular, Yoon et al. studied the magnetic behavior of Li-ethylenediamine-reduced Ti_3_C_2_T_x_ powders and showed that their powders are Pauli paramagnetics above 10 K, with a temperature-dependent Curie term below this threshold [46].

## 4. Polysaccharide Composites Encompassing MXene

### 4.1. Chitosan Composite of MXene 

Chitosan (CS) is a marine polysaccharide extracted from chitin where it is naturally found in the cell wall of fungi. Chitosan is created via deacetylation of chitin [25,57,58,59]. It is a linear biopolymer encompassing D-glucosamine and N-acetyl-D-glucosamine connected by a β-(1,4) bond with a molecular weight between and 300 and 1000 kDa [60]. Natural polymer composites, among the many attempts to introduce MXene into polymers, are ecologically important because of their biodegradability and biocompatibility, leading researchers to focus on natural amino polysaccharides, such as chitosan (CS). CS is made via the deacetylation of chitin present in insects and crustaceans, and it has antibacterial, antioxidant and hypoglycemic properties, as well as the potential to scavenge cholesterol and triglycerides. Due to their molecular mass and extent of acetylation, natural polymer properties such as solubility, material processing ability, biodegradability, etc. (Table 4), are pursued in the food, agricultural and environmental engineering sectors worldwide [57,59,61,62].

**Figure 3:**
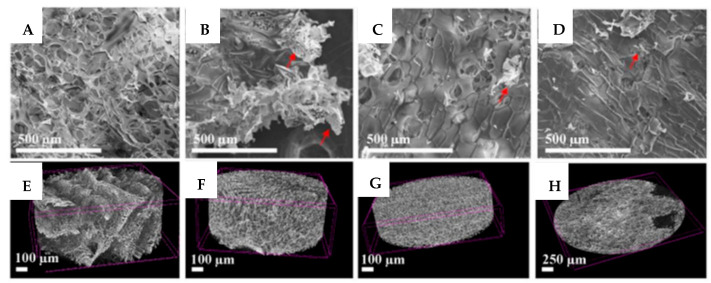
SEM and micro-CT images for the (**A**,**E**) reference CH/SHA matrix nanocomposite and modified with (**B**,**F**) 1 wt.%, (**C**,**G**) 5 wt.% and (**D**,**H**) 10 wt.% of 2D Ti_3_C_2_T_x_ MXene lakes [69].

Lin et al. used a layer-by-layer (LbL) technique to create a MXene/chitosan nanocoating for flexible polyurethane foam (PUF). MXene (Ti_3_C_2_) ultra-thin nanosheets were created by etching Ti_3_AlC_2_ and then exfoliating it (Figure 3). By alternately submerging the PUF in a chitosan solution and a Ti_3_C_2_ aqueous diffusion, the MXene/chitosan nanocoating was deposited, resulting in varying numbers of bilayers (BL) ranging from 2, 5 and 8. The weight gain for the 8 BL coating of PUF was only 6.9% because to the use of ultra-thin Ti_3_C_2_ nanosheets, which minimized the negative influence on the intrinsic attributes of PUF. PUF’s flammability and smoke emissions were dramatically reduced by the Ti_3_C_2_/chitosan coating [69].

**Figure 4:**
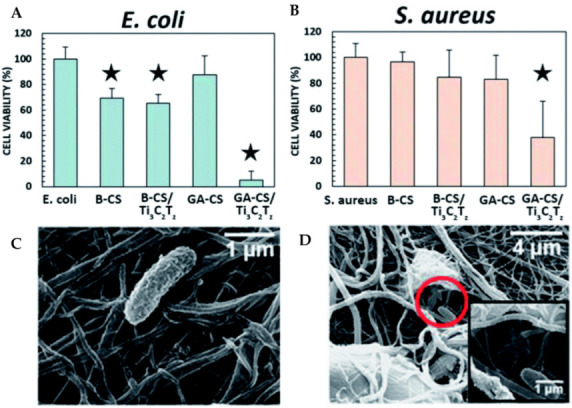
(**A**) *E. coli* and (**B**) *S. aureus* antibacterial activity B-X and GA-X are mats that have been treated with NaOH and glutaraldehyde, respectively. On the 0.75 wt.% Ti_3_C_2_Tz/CS nanofiber mat, SEM micrographs reveal (**C**) undamaged and (**D**) destroyed *E. coli* bacteria. The star symbol denotes samples that differ substantially from the control, *p* ≤ 0.05 [63].

Mayerberger and co-workers produced captured delaminated Ti_3_C_2_T_x_ (MXene) flakes inside chitosan nanofibers using an electrospinning process for application in passive antibacterial wound dressings (Figure 4). After 4 h of treatment with nanofibers loaded with 0.75 wt.% Ti_3_C_2_T_x_, in vitro antibacterial studies were undertaken for crosslinked Ti_3_C_2_T_x_/chitosan composite fibers against Gram-negative *Escherichia coli* (*E. coli*) and Gram-positive *Staphylococcus aureus* (*S. aureus*), and a 95% and 62% reduction in colony-forming units were found, respectively [63].

### 4.2. Cellulose Composite of MXene 

Cellulose is an abundant natural biomaterial and consists of glucose monomers which are exclusively linked by β-(1,4) bonds. Hybrid MXene/cellulose nanofiber (CNF) foam was generated using vacuum filtration, hydrazine-induced foaming and assembly in a piezoresistive pressure sensor using a flexible PI substrate, electrodes made of copper wire, polyimide (PI) and polypropylene (PP) film [70]. The pressure sensor has a high sensitivity across a wide linear range, measuring 419.7 kPa^−1^ for pressures less than 8.04 kPa and 649.3 kPa^−1^ for values between 8.04 and 20.55 kPa. It also has a low detection limit of 4 Pa, a quick response/recovery time of 123/139 ms and a high durability of more than 10,000 cycles. Furthermore, because of the two different types of plane-to-plane interaction in assorted scales produced by the unique porous cellular structure, the sensing performance of the MXene/CNF-foam sensor has been vastly upgraded compared to other piezoresistive pressure sensors.

Hu et al. developed a superior thermally/electrically conductive MXene/cellulose nanocomposite paper via an easy dip-coating protocol. Profiting from the 3D interconnected MXene (Ti_3_C_2_T_x_) network, the ensuing paper revealed an excellent electrical conductivity of 2756 S/m at a nanosheet loading of 1.89 vol% [71]. Subsequent to a polydimethylsiloxane (PDMS) covering, the as-synthesized free-standing nanocomposites with 0.2 mm thickness can attain an exceptional EMI shielding efficiency of over 43 dB in the X and Ku bands at the Ti_3_C_2_T_x_ payload of 1.07 vol%, with no apparent decline being observed after 2000 bending–releasing cycles in the durability assessment. In addition, an in-plane thermal conductivity of 3.89 W/(m⋅K) was attained, ~540% higher than that of PDMS-layered plain filter paper. This study offers a facile strategy for large-scale and greener fabrication of flexible and multifunctional EMI materials [71].

### 4.3. Hyaluronic Acid Composite of MXene 

Hyaluronic acid (HA) is a unique hydrophilic, water-soluble polysaccharide comprising D-glucuronic acid and *N*-acetyl-D-glucosamine, which are connected by β-(1,4) glycosidic bonds [72].

Zhou et al. created a 2D structure of Ti_3_C_2_T_x_ MXene with good conductivity, biocompatibility and antibacterial properties, which they incorporated in the construction of MRSA (methicillin-resistant *Staphylococcus aureus*)-infected wound-healing multifunctional scaffolds (HPEM) [73]. HPEM scaffolds were generated by combining poly(glycerol-ethylenimine) nanosheets, Ti_3_C_2_T_x_ MXene@polydopamine (MXene@PDA) nanosheets and oxidized hyaluronic acid (HCHO). HPEM scaffolds demonstrated multifunctional capabilities such as electrical conductivity, self-healing, tissue adhesion and antibacterial activity, particularly for MRSA resistant to several regularly used antibiotics (antibacterial efficacy was 99.03%), as well as quick hemostatic capability.

### 4.4. Sodium Alginate Composite of MXene 

Alginate is a native polysaccharide acquired from algal and bacterial resources, with brown algae being the marketable supply of alginate [74,75]. It is a linear anionic polymer made up of saccharide units of β-D-mannuronic acid and L-gluronic acid [76].

**Figure 5:**
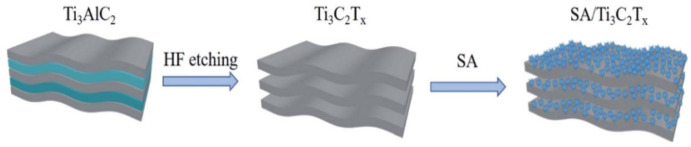
Schematics for the synthesis of MXene/alginate composites [66].

Figure 5 depicts the MXene/alginate composites’ preparation method. The original Ti_3_AlC_2_ (MAX) morphology is uneven (Figure 5). Ti_3_C_2_T_x_ (MXene) exhibits a characteristic two-dimensional organ-like shape after etching with hydrofluoric acid (Figure 5). When applied to Ti_3_C_2_T_x_, alginate first occupies the interlayer before covering a section of the Ti_3_C_2_T_x_ surface. The surface of the MXene/alginate composites is rougher than that of pure Ti_3_C_2_T_x_ (Figure 5), providing a better environment for Pb^2+^ and Cu^2+^ adsorption. Dong et al. developed novel alginate/MXene composites for removal of Pb and Cu ions from wastewater. The alginate/MXene composites utilized in this study enhanced not only Pb and Cu ion chelation, but additionally increased the efficiency of ion transport (Figure 5). Due to the benefits of higher adsorption capacity and shorter equilibrium time, the alginate/MXene composites could accomplish the maximum adsorption capacity for Pb^2+^ and Cu^2+^ at 382.7 and 87.6 mg/g, respectively, and attain adsorption equilibrium within 15 min. The composites developed in this work have the potential to lead the way for novel approaches for designing adsorbents with high adsorption capacity and efficiency.

**Figure 6:**
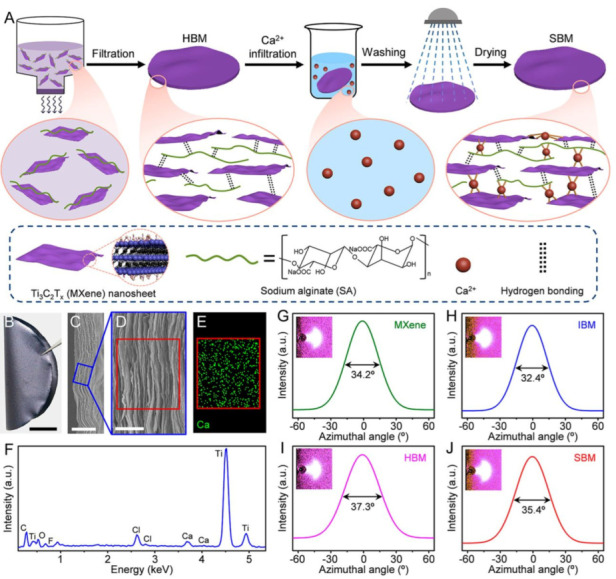
SBM sheet fabrication and structural characterization. (**A**) Vacuum filtering was used to assemble the MXene-SA hybrid building blocks into an HBM sheet. The Ca^2+^ was subsequently absorbed by the HBM sheet, resulting in the formation of an SBM sheet. (**B**) A photograph of an SBM sheet demonstrating its pliability. (**C**) A low-resolution SEM picture of the SBM sheet’s fracture surface. (**D**) A high-resolution SEM picture of the region indicated in (**C**). (**E**) Ca^2+^ EDS mapping and (**F**) EDS spectra of the region delineated in (**D**). WAXS patterns for an incident Cu-K X-ray beam parallel to the sheet plane and related azimuthal scan profiles for the 002 peak for the sheets: (**G**) MXene, (**H**) IBM, (**I**) HBM and (**J**) SBM. Scale bars: (**B**) 1 cm, (**C**) 5 μm and (**D**) 1 μm [77].

Wan et al. established the fabrication of high-performance MXene-based sheets via a sequential bridging approach, in which MXene nanosheets are first hydrogen-bonded to SA, and then the resulting hybrid MXene-SA building blocks are ionic-bonded to calcium ions (Ca^2+^) (Figure 6). The resulting sequentially bridged MXene (SBM) sheets exhibit a high in-plane tensile strength of 436 MPa, outstanding toughness of 8.39 MJ/m^3^ and a good Young’s modulus of 14.0 GPa, which are 6.9, 13.5 and 2.5 times higher than pure MXene sheets [77].

## 5. Biomedical Applications of MXenes

With their unique 2D layered structure and high physical and chemical properties, including hydrophilicity, biocompatibility, light-heat conversion performance and mechanical flexibility [27], MXenes can be used in a variety of biomedical applications, including sensors [78], bioimaging [79], tissue engineering [10], photothermal therapy [80], antibacterial photothermal therapy [81], antibacterial therapy and drug delivery systems [10,82], among others.

**Figure 7:**
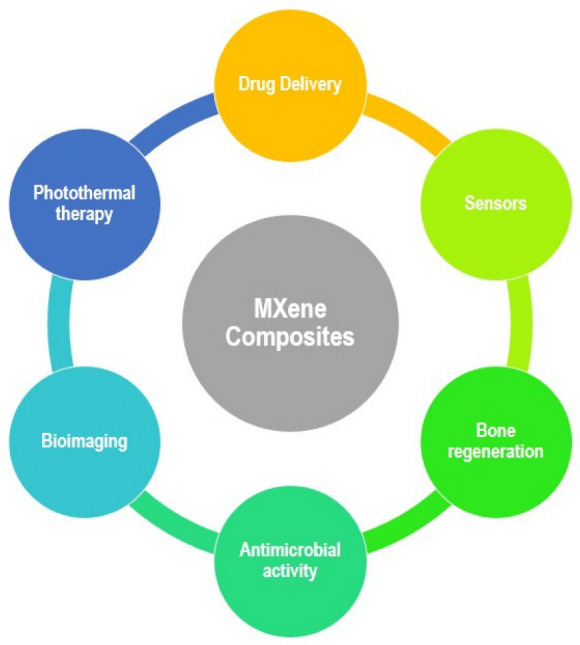
Schematic representation of assorted applications of MXene composites.

MXenes’ diverse and attractive features make them an ideal candidate for wide-ranging applications. They are characterized by exceptional properties, such as high Young’s Modulus, unique morphology and electrical conductivity, and this makes them an excellent choice for a wide range of technologies, including catalysis, sensors and energy storage. Throughout many cases, including electromagnetic interference shielding, MXenes outperform many currently available alternatives. The most important biological uses of MXene composites are depicted in Figure 7 [83].

MXene hydrogels have shown great promise for a variety of in vivo biological applications, including cancer treatment and drug delivery. MXene hydrogels offer the following advantages: (1) High wettability, which facilitates the dispersion and stability of MXene-derived photodynamic and photothermal agents in physiological media. (2) Thanks to the polar end groups, anti-cancer medicines can be easily grafted onto MXene surfaces. (3) By fine-tuning the swelling performance of MXene hydrogels, excellent anticancer drugs with loading capacities as high as 84% and high release percentages can be obtained.

### 5.1. Drug Delivery 

Conventional cancer treatment methods, such as chemotherapy and photodynamic therapy (PDT) [25,83], can harm nonmalignant cells as well as malignant ones. The development of stimuli-responsive materials capable of identically sensing the comparatively lower pH of tumor cells [84] has the potential to greatly alleviate this problem [85]. Several studies have been conducted in recent years to establish an appropriate nanoplatform for drug carrier applications [86,87]. According to their intrinsic pH sensitivity and excellent photothermal conversion, MXenes provide the cumulative photothermal ablation and tailored medication release impact. Xing et al. reported cellulose and Ti_3_C_2_ MXene composite hydrogels [23], which when the anticancer agent is loaded doxorubicin, could accomplish faster release of the drug (DOX). When triggered by irradiation with an 808 nm light, light-induced enlargement of the pores inside the three-dimensional cellulose-based networks occurs. The scientists revealed that the combination of photothermal therapy (PTT) and extended adjuvant chemotherapy utilizing this nanoplatform was very efficient for simultaneous tumor elimination and tumor recurrence suppression, implying that it has the potential to grow into an efficient theranostics system in the future.

Ti_3_C_2_-based nanoplatforms for synergistic PTT, PDT and chemotherapy were developed by Liu et al. [80,81]. When irradiated with an 808 nm laser, the produced Ti_3_C_2_-based nanosheets showed an excellent extinction coefficient value of 28.6 Lg^−1^·cm^−1^, an amazing efficiency for photothermal conversion of around 58.3% and effective creation of singlet oxygen [81]. Doxorubicin (DOX), a chemotherapeutic drug, was placed in MXene with a hyaluronic acid coating applied to its surface (HA) to boost its biocompatibility. This also improved the selectivity towards malignant cells identified by the CD44 antigen, allowing for active targeting. In vitro and in vivo investigations have been performed, which uncovered that Ti_3_C_2_-DOX exhibits better biocompatibility, as well as tumor-specific accumulation behavior and drug-releasing ability in response to stimuli, and could be deployed in PTT/PDT/chemotherapy to destroy malignant cells and tumor tissues [88].

A highly elastic nanocomposite (NC) colloidal gel was manufactured in a factory using acrylamide’s in-place radical chemical action [32]. In particular, the authors used an exfoliated two-dimensional MXene nanosheet-based Ti_3_C_2_ as a crosslinking agent instead of traditional organic crosslinkers. NC hydrogolds presented enhanced mechanical properties with tensile strengths of 66.5 to 102.7 kPa, compressive strengths of 400.6 to 819.4 kPa and elongation of 2158.6% to 3047.5%. NC hydrogels exhibited good sustained-release performance, higher drug loading amounts (97.5–127.7 mg/g) and higher percentage releases (62.1–81.4%), greatly superior to those of the BIS/PAM hydrogel. The enhanced mechanical performances can be attributed to the honey-comb-like fine structure with uniform pores as well as more flexible polymer chains [32].

### 5.2. Sensors

MXenes are appealing for sensors due to their excellent conductivity and general-purpose surface chemistry. Sharma et al., however, reported the simple fabrication of a highly sensitive and robust capacitive pressure sensor for ultra-low-pressure measurement using MXene (Ti_3_C_2_T_x_)/poly(vinylidene fluoride-trifluoroethylene) (PVDF-TrFE) nanofibrous composite scaffolds as a dielectric layer between biocompatible poly-(3,4 ethylenedioxythiophene) polystyrene. The sensor has a high sensitivity of 0.51 kPa^−1^ and a low detection limit of 1.5 Pa [89]. Furthermore, it offers linear sensing over a large pressure range (0–400 kPa) as well as strong dependability over 10,000 cycles, even at extremely high pressure (>167 kPa). MXene loading improves the sensitivity of the nanofiber-based sensor by increasing the dielectric constant to 40 and decreasing the compressive modulus to 58% when compared to pristine PVDF-TrFE nanofiber scaffolds. The suggested sensor may be used to detect a patient’s health status by monitoring physiological data (pulse rate, breathing, muscle movements and eye twitching), and it is also a good candidate for a future generation human–machine interaction device [89].

As a result, the Au/MXene nanocomposite described in this paper might be used as an electrochemical transducer in electrochemical biosensors [90].

### 5.3. Photothermal Therapy (PTT) 

Radiation and chemotherapy are currently quite often used as cancer treatment procedures. However, these approaches are not highly focused and can cause harm to normal tissues in addition to cancer cells, resulting in serious side effects. Improved selectivity thereby decreases the adverse effects by using light-controlled treatments such as PTT [5]. Photothermal agents are delivered into cancer tissues, where they convert the light energy to heat energy. Cancer cells, in general, have poor heat resistance, therefore when heat is generated, these cells are eliminated. Since visible light has a poor tissue permeation, near-infrared (NIR) radiations are often employed in PTT. There are two classifications of NIR based on the wavelength of radiation: (1) the first NIR bio-window, which has a wavelength range of 750 to 1000 nm, and (2) the second near-infrared bio-window, which has a wavelength range of 1000 to 1350 nm. According to studies, the second NIR bio-window has several benefits over the first one, such as a lower required laser penetration depth and a higher maximum allowable exposure (MPE). Consequently, its uses are restricted due to a scarcity of materials with effective NIR light absorption and photothermal conversion ability. MXene-based materials are shown to be more efficient in both bio-windows, which is a tremendous benefit. In addition, MXenes’ vast surface area provides anchoring sites and enables an effective build-up of toxins in tumor cells while undergoing cancer therapy. 

Hussein et al. developed multiple Ti_3_C_2_T_x_-based 2D plasmonic nanocomposites (Au/MXene and Au/Fe_3_O_4_/MXene) with comparable anti-cancer photothermal treatment (PTT) capabilities, but with lower in vivo toxicity than pure MXene. Morphological evaluation using XRD, SEM and TEM demonstrated that Au/MXene and Au/Fe_3_O_4_/MXene were effectively synthesized. In vitro, both novel composites demonstrated a significant dose-dependent PTT impact against MCF7 human breast cancer cells. In vivo acute toxicity experiments utilizing zebrafish embryos revealed that Au/MXene and Au/Fe_3_O_4_/MXene exhibited lower embryonic mortality (LC_50_ 1000 g/mL) than pure MXene (LC_50_ = 257.46 g/mL) [91].

Szuplewska et al. [92] demonstrated the performance of the photothermal treatment approach employing “Ti_2_C-PEG” by assessing the exposed cells to progressively higher quantities of the tested substance under 808 nm laser irradiation for 2 min.

**Figure 8:**
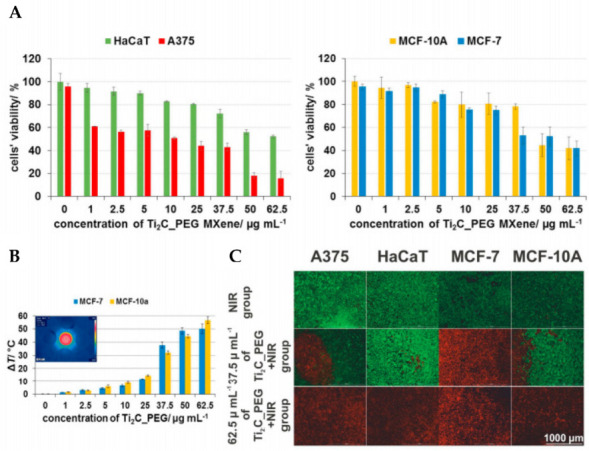
In vitro PTT efficacy (**A**) relative viabilities of HaCaT, A375, MCF-10A and MCF-7 cells after the PTT procedure with the use of various concentrations of Ti_2_C_PEG flakes. (**B**) Comparison of the efficacy of 2D Ti_2_C-PEG as a novel PTT agent with the measured temperature changes for cancer and non-cancer cells on the example of human mammary gland-derived cell lines. (**C**) Exemplary microscopic images after PTT treatment, including the NIR group and groups exposed to various concentrations of the test material and NIR [92].

The effective death of malignant cells was observed after 24 h of incubation with 2D Ti_2_C and additional NIR laser irradiation. In comparison, non-malignant cells survived the PTT treatment with a viability of more than 70%, even at a concentration of 37.5 g/mL (Figure 8a,b). The chemical composition of the surface and the “Ti_2_C-PEG” flakes’ relatively small planar dimension may result in strong MXene affinity for cell membranes, resulting in successful endocytosis into the cells and nanomaterial dispersion primarily inside the cellular cytoplasm. The ability of delaminated “Ti_2_C-PEG” to convert light to thermal energy results in an increase in intracellular temperature (up to 50 °C for MCF-7 cells treated with 62.5 g·mL^−1^) and subsequent ablation of cancer cells after laser irradiation (Figure 8c) [92].

Lin et al. developed an Nb_2_C-PVP hybrid photothermal agent to achieve in vivo photothermal ablation of tumor xenografts in mice with great efficiency at frequencies matching both bio-windows. A digital caliper was used to measure the tumor volume in six groups of mice every two days. It was revealed that the tumor-bearing areas of the control mice remained large after 16 days of various treatments. Similarly, the tumor-containing regions of Nb_2_C-PVP + NIR-I and Nb_2_C-PVP+NIR-II animals were totally eliminated [86].

**Figure 9:**
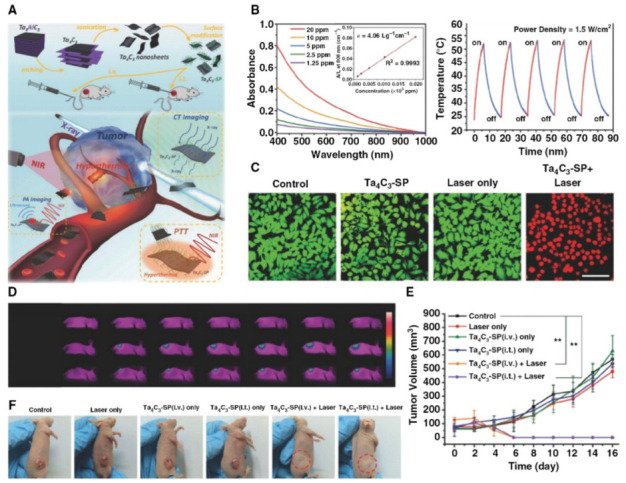
Ta_4_C_3_-SP MXene nanosheets have photothermal treatment effects both in vitro and in vivo. (**A**) Schematic representation of Ta_4_C_3_-SP MXene nanosheets employed in PTT. (**B**) Ta_4_C_3_-SP MXene nanosheet absorbance spectra and photothermal stability after five heating and cooling cycles. (**C**) Confocal fluorescence imaging of in vitro photothermal ablation of 4T1 cells following multiple treatments at 1.5 W cm^−1^ (scale bar: 100 m). (**D**) Infrared thermal pictures at the tumor site of 4T1 tumor-bearing mice in control, Ta_4_C_3_-SP (i.v.) + NIR laser and Ta_4_C_3_-SP (i.t.) + NIR laser groups at different time intervals during laser irradiation. (**E**) Tumor growth curves following various treatments. (**F**) Images of tumor-bearing mice following PTT [80].

It was discussed by Huang et al. that although Ti_3_C_2_-SP nanosheets have a high PCE (30.6%) when used as photothermal agents, a recent study found that Ta_4_C_3_-SP nanosheets had a higher PCE (44.7%) when exposed to an 808 nm laser and have been used in PTT (Figure 9A) [80]. These biomaterial Ta_4_C_3_-SP nanosheets have a sheet-like shape, a lateral dimension of 100 nm and high light absorption over a wide wavelength range (Figure 9B). After 5 min of laser irradiation (1.5 W cm1), the temperature may quickly rise to around 55 °C. Furthermore, the Ta_4_C_3_-SP nanosheets demonstrate good thermal stability even after five heating and cooling cycles (Figure 9B). Without laser irradiation, cell cytotoxicity revealed that Ta_4_C_3_-SP nanosheets had no influence on the survival of 4T1 cells, even at concentrations as high as 400 µg·mL^−1^, indicating their complete biocompatibility. The photothermal performances are affected by laser power density, which was proven by confocal fluorescence imaging after various treatments (Figure 9C). Ta4C3-SP nanosheets may immediately collect in tumors after intravenous (i.v.) or intra-tumoral (i.t.) treatment in a mouse model, and tumor temperatures swiftly climbed to 60 °C (i.v.) or 68 °C (i.t.) from 30 °C after 6 min of 808 nm laser irradiation (Figure 9D). Ta_4_C_3_-SP nanosheets may accomplish excellent photothermal elimination of tumors following irradiation with an 808 nm laser, as shown in Figure 9E. Figure 9F depicts photographs of 4T1 tumor-bearing mice 16 days after different treatments, confirming the good photothermal ablation potential of Ta_4_C_3_-SP nanosheets for tumors [80].

### 5.4. Bioimaging

Bioimaging has sparked interest in early cancer detection because of the capacity to provide in-depth insights into biological activities as well as a wide variety of diagnostic markers [79]. Due to its user-friendliness and low-cost equipment, fluorescence microscopy has become one of the most common bioimaging modalities. Though MXenes have a relatively small luminosity in aqueous solutions, it has been discovered that attaching fluorescent molecules to their surface increases the brightness. The X-ray computed tomography (CT) scan is another significant bioimaging platform that facilitates the usage of MXene. Traditional CT contrast agents, such as iodine-containing compounds, had a poor blood circulation rate, the possibility for renal impairment and severe toxicity, necessitating the development of efficient and biocompatible materials. Tantalum-rich MXenes, such as Ta_4_C_3_, have been discovered to be suitable for use as CT imaging contrast agents due to their high biocompatibility, proper size and environmentally friendly manufacture [93]. MRI is another outstanding method, like CT, that may be utilized for bioimaging in patients. It is being used as a solution for people who have allergic responses to CT contrast agents. Materials containing gadolinium, which are extensively employed as MRI contrast agents, have been linked to an increased risk of kidney injury [8]. As an MRI contrast agent, the composites made by growing nanosized manganese oxide on MXenes have shown great promise [79].

### 5.5. Bone Regeneration 

According to recent research, MXene-based composites might be used in guided bone regeneration (GBR), which is often used in oral rehabilitation procedures such as dental surgical treatment and periodontal regeneration to protect healed bones from soft tissue interference [83]. 

Chen et al. created Ti_3_C_2_T_z_-improved poly(lactic acid) (PLA) membranes by employing n-octyltriethoxysilane as an interfacial mediator (OTES) [94]. The addition of these Ti_3_C_2_T_x_ nanosheets increased the membranes’ biocompatibility, cell adhesion, osteogenic differentiation and proliferation. These nanocomposite membranes were strong and biocompatible, indicating that they might be used as the GBR membrane [94].

**Figure 10:**
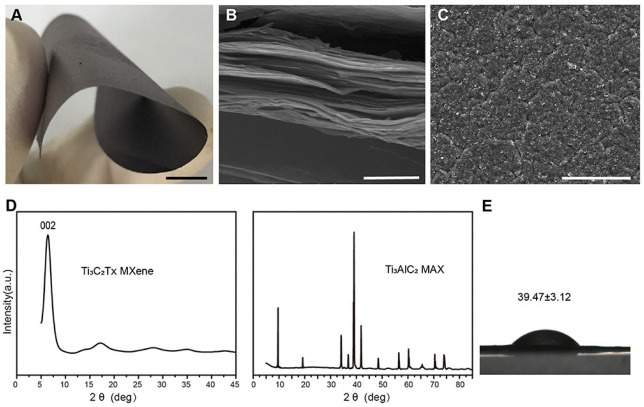
Multilayered Ti3C2Tx MXene film characterization. (**A**) Photograph of the MXene film, which is flexible and free-standing. (**B**) SEM picture of a cross-section of MXene sheets, scale bars: 5 mm, 1 μm. (**C**) SEM picture of the surface of MXene films. Scale bars are 50 μm long. (**D**) Ti_3_C_2_T_x_ MXene and Ti_3_AlC_2_ MAX XRD patterns. (**E**) Water contact angles (*n* = 3) on Ti_3_C_2_T_x_ MXene films [96].

Zhang et al. [95] synthesized Ti_3_C_2_T_x_ MXene, wherein the flexible free-standing multilayered Ti_3_C_2_T_x_ MXene film could be readily peeled from the membrane filter after etching and drying (Figure 10A). The MXene film measured around 50 mg and was roughly 40 m-thick, as characterized by X-ray diffraction (XRD) and scanning electron microscopy (SEM), affirming the multilayered MXene structure. The dried sample’s cross-section SEM picture revealed a distinctive multilayered stacked structure (Figure 10B), and the surface appearance was rough and chaotic (Figure 10C). Rough-surfaced materials are ideal for cell adhesion, proliferation and bone cell differentiation. Furthermore, Ti_3_C_2_T_x_ MXene’s thick multilayered stacking structure makes it an efficient obstacle for a GBR membrane to prevent fibroblast migration. The (Ti_3_C_2_T_x_) XRD spectra (Figure 10D) demonstrated the elimination of Ti_3_AlC_2_ peaks and the presence of just one intense (002) peak. The XRD analysis confirmed earlier findings, revealing that the Ti_3_C_2_T_x_ MXene was effectively etched and produced. The water contact angle on MXene films was 39.47° (3.12°) according to a drop-shape investigation (Figure 10E). Due to the functional groups on the Ti_3_C_2_T_x_ surface, the decreased water contact angles imply that the surface of MXene films is hydrophilic. According to several studies, this hydrophilicity increases cell adhesion and cell spreading. These features point to MXene’s potential for use in GBR treatment [95].

### 5.6. Antimicrobial Activity 

**Figure 11:**
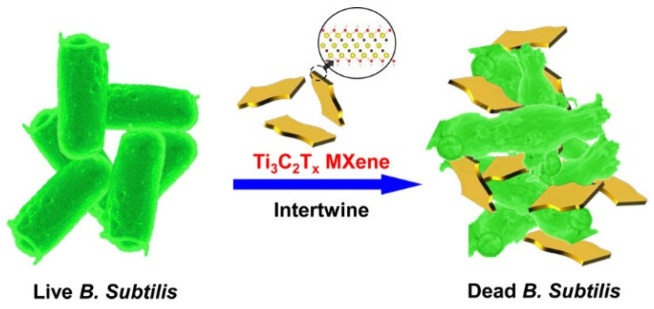
Schematic depiction of antibacterial activity of Ti_3_C_2_T_x_ MXene [3].

Nanomaterials with two dimensions and polymers have been used and investigated for their antimicrobial utility [96,97]. Among them, MXene materials have demonstrated more antibacterial action than graphene oxide (GO), a well-studied antimicrobial agent, and Ti_3_C_2_ has demonstrated considerably stronger antibacterial activity against both *E. coli* and *B. subtilis* [3]. The precise role of MXenes’ antibacterial action is uncertain. The most plausible hypotheses are that: (1) the sharp edges of Ti_3_C_2_ MXenes allow for effective absorption on the surface of microorganisms, (2) the sharp edges of MXenes can damage the microbial membrane (Figure 11) and (3) MXenes can react with biomolecules in the cytoplasm and cell wall, causing cell microstructure breakdown and thus bacterial species’ death [98]. 

**Figure 12:**
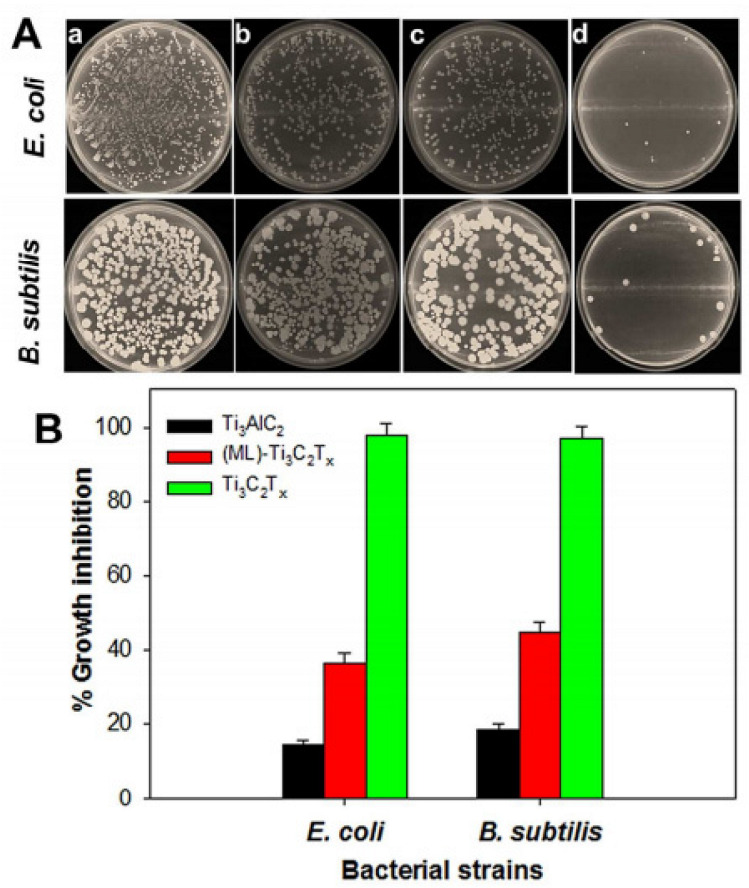
(**A**) Photographs of agar plates onto which *E. coli* (**top panel**) and *B. subtilis* (**bottom panel**) bacterial cells were re-cultivated after treatment for 4 h with a control (**a**), and 100 µg/mL of Ti_3_AlC_2_ (**b**), ML-Ti_3_C_2_T_x_ (**c**) and delaminated Ti_3_C_2_T_x_ (**d**). (**B**) Percentage of growth inhibition of bacterial cells treated with 100 µg/mL of Ti_3_AlC_2_, ML-Ti_3_C_2_T_x_ and delaminated Ti_3_C_2_T_x_ [3].

Rasool et al. tested the antibacterial efficacy of three materials (Ti_3_AlC_2_ (MAX), as-produced ML-MXene and delaminated Ti_3_C_2_T_x_ nanosheets) against *E. coli* and *B. subtilis* to see how delamination affects MXenes’ antibacterial efficacy. The colony counting method was used to measure the bacterial growth inhibition. Figure 12A shows pictures of agar plates onto which control and bacterial cells were re-cultivated after being exposed to the same concentration of 100 µg/mL of nanomaterial for 4 h. The percentage growth inhibition of both bacterial strains exposed to the materials under research is depicted in Figure 12B. For *E. coli* and *B. subtilis*, MAX dispersion only inhibited growth by 14.39% ± 1.43% and 18.34% ± 1.59%, respectively. The antibacterial activity of the ML-Ti_3_C_2_T_x_ dispersion was somewhat greater than that of MAX, with *E. coli* and *B. subtilis* growth suppression of 30.55% ± 2.56% and 33.60% ± 2.89%, respectively (Figure 12B). Therefore, when cells are exposed to a colloidal solution of delaminated Ti_3_C_2_T_x_ MXene, the vitality of *E. coli* and *B. subtilis* cells rises to 97.70% ± 2.87% and 97.04% ± 2.91%, respectively, indicating substantially higher inhibition [3]. The antibacterial activity of the three materials against both bacterial strains differed significantly, where delaminated Ti_3_C_2_T_x_ MXene, for instance, had substantially stronger antibacterial activity than MAX and MLTi_3_C_2_T_x_ MXene and could be explored in future experiments.

**Figure 13:**
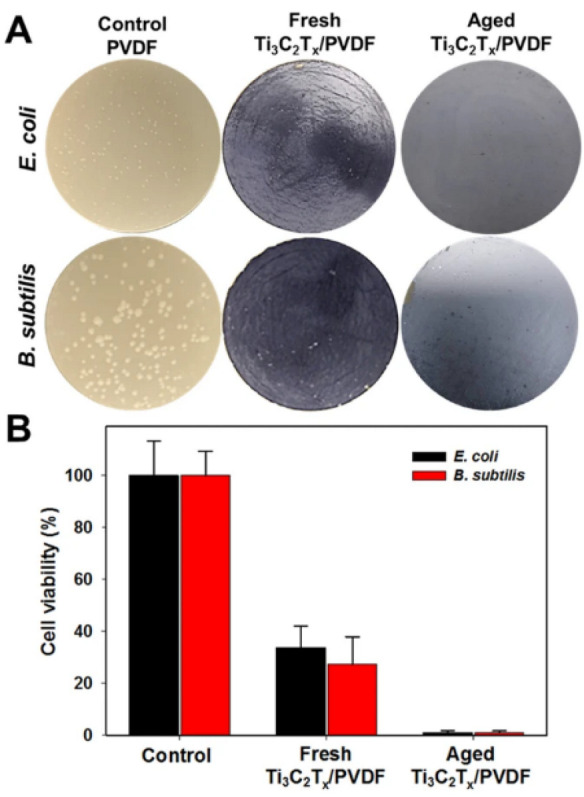
Ti_3_C_2_T_x_ MXene has antibacterial action. (**A**) Photographs of *E. coli* and *B. subtilis* growth on unmodified PVDF (control), fresh and aged Ti_3_C_2_Tx MXene-coated PVDF membranes cultured for 24 h at 35 °C. (**B**) Cell viability measurements of *E. coli* and *B. subtilis* treated with Ti_3_C_2_T_x_, Fresh Ti_3_C_2_T_x_/PVDF and Aged Ti_3_C_2_T_x_/PVDF. The colony-forming count method was used to calculate survival rates. Reproduced with permission from [3].

In another investigation [99], the same research group created micrometer-thick MXene (Ti_3_C_2_T_x_)-based membranes with antibacterial characteristics. When fresh MXene (Ti_3_C_2_T_x_)-based membranes were compared to a control polyvinylidene fluoride (PVDF) membrane, the antibacterial rate was 67% against *E. coli* and 73% against *B. subtilis*. Remarkably, the aged MXene (Ti_3_C_2_T_x_)-based membranes could limit bacterial growth by more than 99%. According to the flow cytometry results, 70% of both bacteria were killed after 24 h of exposure to the membranes (Figure 13) [3].

## 6. Toxicity of (Ti_3_C_2_T_x_) MXene 

The biological qualities of MXenes are related to their carbon and/or nitrogen content, which are the fundamental building blocks of all living creatures. While early transition metals such as Ti, Ta and Nb are considered largely harmless, accumulating evidence suggests that they may be hazardous. As a result, in-depth research shows that exploring easy, low-cost and environmentally friendly techniques to limit their potential toxicity is highly valued. MXene-based nanocomposites with exceptional properties such as tunable morphologies/structures, biocompatibility, remarkable physiological stability, biodegradability and simple functionalization procedures could be employed in a range of clinical and biological applications, as these characteristics are common obstacles for most organic substances. Toxicity, biosafety and biocompatibility concerns, on the other hand, should be thoroughly investigated for these 2D MXenes as well as critical factors including solubility, dispersibility and long-term toxicity [51]. In one study, Alhussain et al. looked at the potential toxicity of MXene nanosheets at the early stages of embryogenesis as well as angiogenesis and discovered that they may have a negative effect on the primary period of embryogenesis, where approximately 46% of MXene-exposed embryos died 1–5 days after exposure [100,101].

## 7. Conclusions and Perspective

In recent years, MXenes have shown great promise in biological applications. This review focused on recent breakthroughs in the design and fabrication of pure and functionalized MXenes and their composites, with emphasis on biological and biomedical applications, among others. Due to their diverse chemistries, distinct morphologies and outstanding electrical conductivities, they exhibit attractive prospects for biological applications. They provide excellent composites with better properties when coupled with polymers, ceramics, metals and carbon-based nanocomposites. Furthermore, the synthesis procedures are scalable and reasonably priced, making them more enticing. To generate consistent terminations with the same sort of surface entities, several methods must be investigated. Theoretical studies have previously revealed that bare MXene outperforms surface-terminated MXene in many applications; hence, safer methodologies for the synthesis of bare MXene must be devised in order to significantly increase its usefulness. Though numerous studies on MXene applications have been conducted, more attention should be aimed at understanding the underlying concepts and basics, which can aid in the development of more efficient materials. These studies should lead to a rapid increase in the synthesis of a new family of MXenes, as well as a promising future for them in biological devices. This review focused on MXenes, their derivatives and MXene-based composites in biosensors, cancer theranostics, drug delivery to cancer biomarkers and antimicrobial activities. We believe that the use of MXenes in biomedical research is in its infancy and that rigorous guidelines are needed before MXene-based products can be used in biomedicine. Finally, despite the large number of MXene-polymer composites generated, MXene-metal- or ceramic-based mixes are still in the initial phases, and actions must be taken to understand their behavior, from their microstructural features to their material physical properties.

## Figures and Tables

**Table 1:** MXenes’ main mechanical, electrical, magnetic and optical behavior [10].

Properties	Remarks
Mechanical properties	MXenes mechanical characteristics are primarily determined by the surface endings. MXenes with an O ending display high stiffness, while the materials with OH^−^or F^−^ terminations exhibit lower elastic rigidity [11].
Optical properties	MXenes have robust plasmonic resonances, a broad optical transparency space, nonlinear optical performance, optical transparency, photothermal conversion, tunable optical responsiveness and resilient surface-sensitive optical features. Changing the functional groups on the surfaces of MXenes can alter their optical properties.
Electronic features	Mostly, 2D materials belong to the nonmagnetic type, which limits their spintronics applications; however, some MXenes, namely Ti_2_N, Cr_2_C and Ti_2_C, are ferromagnetic, while others such as Cr_2_N and Mn_2_C are antiferromagnetic. Surface functionalization can easily change the magnetic attributes of MXenes.
Magnetic properties	MXenes possess several appealing electrical properties, including semi-conductivity, topological insulativity and metallicity. MXenes’ conductivity is mostly governed by the manufacturing and delamination processes utilized.

**Table 2:** An overview of MXene hydrogel products.

Composition	Preparation Time	Preparation Temperature	Stability	Mechanical Property	Conductivity	Photothermal	Application	Ref.
Mxene/PVA/PAM	>6 h	90 °C	Moderate	Up to 1200%	Up to 4.25	-	Sensors	[30]
MXene/Cellulose	>1.5 h	65 °C	Moderate	Compressive strength 34.7 KPa	-	Yes	Photothermal therapy	[23]
MXene/PNIPAM	Few minutes	20 °C	Great	Storage modulus 3: 5 KPa	0.019 S/m	Yes	Smart «window»	[31]
MXene/PAM	-	20 °C	Great	Up to 3047.5%	-	-	Drug release	[32]
MXene/PNIPAM/PAM	>24 h	10 °C	General	Up to 1400%	1.092 S/m	-	sensor	[33]
MXene/honey/chitosan	~ 1 h	37 °C	Moderate	-	yes	-	Cell attachment and survival	[34]
MXene/PVA	5 min	20 °C	Moderate	3400%	Yes	-	Sensors (GF: 25)	[35]
MXene/PVA	>9.5 h	90 °C	Moderate	1200%	Yes	-	Sensors (GF: 40)	[36]
MXene/PNIPAM	24 h	20 °C	General	Young’s modulus: 8.66 KPa	-	Yes	Photothermal response smart with sensor	[37]

**Table 3:** Percolation threshold of various MXene polymers.

Polymer	MXene	Processing	Percolation Threshold (Vol%)	Ref.
Polyacrylamide (PAA)	d-Ti_3_C_2_	Solvent	1.7	[47]
Polystyrene (PS)	d-Ti_3_C_2_	Solvent	0.26	[48]
PVDF-TrFE-CFE	d-Ti_3_C_2_	Solvent	6.9	[49]
CoPA	d-Ti_3_C_2_	Solvent	0.05	[50]
Natural rubber	d-Ti_3_C_2_	Solvent	0.91	[51]
Acrylic resin	d-Ti_3_C_2_	Solvent	6.07	[52]
Acrylic resin	PPy-d-Ti_3_C_2_	Solvent	6.4	[52]
Polydimethylsiloxane (PDMS)	d-Ti_3_C_2_	In situ curing	0.7	[53]
Polydimethylsiloxane (PDMS)	d-Ti_3_C_2_	In situ curing	1.3	[54]
Polydimethylsiloxane (PDMS)	HPSi-d-Ti_3_C_2_	In situ curing	1.4	[54]
poly(vinylidene fluoride (PVDF)	d-Ti_3_C_2_	Solvent	6.76	[55]
Polyuethane (PU)	d-Ti_3_C_2_	Solvent/wet spinning	0.28	[56]

**Table 4:** MXene-encompassing polysaccharides.

No.	MXene	Polymer	Application	Result	Ref
1	Ti_3_C_2_T_x_	Chitosan nanofibers	Antibacterial Properties	An electrospinning-based method allows incorporation of delaminated Ti_3_C_2_T_z_ (MXene) flakes into chitosan nanofibers for passive antibacterial dressing uses. After 4 h of treatment with nanofibers loaded with 0.75 wt.% Ti_3_C_2_T_z_, in vitro antibacterial studies on crosslinked Ti_3_C_2_T_z_/chitosan composite fibers against Gram-negative *E. coli* and Gram-positive *Staphylococcus aureus* (*S. au-reus*) showed 95% and 62% reductions in colony-forming units, respectively.	[63]
2	MXene@CeO_2_	Oxidized sodium alginate	Skin multimodal therapy	For multimodal therapy of skin affected by MDR bacteria, researchers created a self-healing, injectable, multifunctional hydrogel scaffold (FOM) based on MXene@CeO_2_ nanocomposites. The MXene@CeO_2_ nanocomposites were incorporated into a dynamic Schiff-centric chemical crosslinked framework of F127-PEI and OSA to create the multifunctional FOM scaffold.	[64]
3	Ti_3_C_2_T_x_	Cellulose acetate	Water purification and biomedical applications	For *E. coli* and *B. subtilis*, a 10% MXene@CA membrane inhibited growth by more than 98% and 96%, respectively. In addition, the optimal membrane’s hydrophilicity (water contact angle = 60.8°) was greatly improved, favoring good antifouling qualities. The described nanofiltration membrane, particularly the 10% MXene@CA version, could be deployed for biomedical and water purification applications.	[65]
4	Ti_3_C_2_T_x_	Sodium alginate (SA)	Lead and copper ion removal from aqueous solutions	Through the advantages of increased adsorption capacity and decreased equilibrium time, the MXene/alginate composites attain a maximum adsorption capacity for Pb^2+^ and Cu^2+^ of 382.7 and 87.6 mg/g, respectfully, and reach adsorption equilibrium within 15 min.	[66]
5	Ti_3_C_2_T_x_	Chitosan (CS)	Biosensor for Detection of Potential Prostate Cancer	MXene interfaced with chitosan provides a suitable scaffold for enzyme localization to manufacture a sensitive biosensor with a lower detection boundary of 18 nM and a linear span up to 7.8 M, according to electrochemical tests, SEM and AFM analyses.	[67]
6	Ti_3_C_2_T_x_	Sodium alginate (SA)	Adsorption of methylene Blue (MB)	According to the findings, Ti_3_C_2_T_x_/SA-30% was chosen as the optimal mass ratio, with a maximum adsorption capability of 92.17 mg/g at pH 7 and 298 K. After three cycles, the MB adsorption efficiency remained at 81.36%.	[68]

## Data Availability

This research did not report any data.
